# De-Skewing LiDAR Scan for Refinement of Local Mapping

**DOI:** 10.3390/s20071846

**Published:** 2020-03-26

**Authors:** Lei He, Zhe Jin, Zhenhai Gao

**Affiliations:** State Key Laboratory of Automotive Simulation and Control, Jilin University, Changchun 130022, China; jlu_helei@jlu.edu.cn (L.H.); gaozh@jlu.edu.cn (Z.G.)

**Keywords:** skewing, slam, 3D-LiDAR, point cloud, sensor fusion

## Abstract

Simultaneous localization and mapping have become a basic requirement for most automatic moving robots. However, the LiDAR scan suffers from skewing caused by high-acceleration motion that reduces the precision in the latter mapping or classification process. In this study, we improve the quality of mapping results through a de-skewing LiDAR scan. By integrating high-sampling frequency IMU (inertial measurement unit) measurements and establishing a motion equation for time, we can get the pose of every point in this scan’s frame. Then, all points in this scan are corrected and transformed into the frame of the first point. We expand the scope of optimization range from the current scan to a local range of point clouds that not only considers the motion of LiDAR but also takes advantage of the neighboring LiDAR scans. Finally, we validate the performance of our algorithm in indoor and outdoor experiments to compare the mapping results before and after de-skewing. Experimental results show that our method smooths the scan skewing on each channel and improves the mapping accuracy.

## 1. Introduction

Various kinds of sensors are becoming increasingly important in a self-driving vehicle and multi-sensor fusion plays an important role in mapping or navigation [1]. They help vehicles or robots perceive the environment [2], recognize objects [3], and localize themselves in a global coordinate system [4]. The accuracy and precision of sensor data have a significant effect on the results of simultaneous localization and mapping (SLAM).

In the field of autonomous driving, LiDAR (light detection and ranging) provides a rich, 3D point cloud in real-time by using a laser and detector pair mounted in a compact housing. If there is not any special instruction, LiDAR in the following section refers specifically to the field of autonomous driving. Furthermore, the rotational speed of LiDAR ranges from 5 to 20 Hz and it can create 360° images with reflected points. As laser has strong power and can hardly be affected by light or other electromagnetic waves, LiDAR has been widely used in pose estimation or environmental reconstruction [5]. However, LiDAR also has weaknesses. Different from camera or IMU working at a high frequency, it suffers from sensing accuracy when vehicles or robots moving fast suddenly. In addition, the laser reflection can be influenced by snow or rain which may lead to distant mistakes. These factors often result in a pulsed or serrated point cloud and noise points. Thus, the mapping system may be struggling when processing point clouds, then leading to tough output eventually.

Various optimization approaches are applied on slam, like the graph-based approach [6], Ceres Solver [7], and GTSAM optimization toolbox [8]. By minimizing the accumulated errors, these methods simplify the computational complexity and avoid reprocessing the mass sensor data during execution. Although the optimization model having been improved increasingly, the errors existing in raw LiDAR point cloud have not been valued greatly. The motion of platform carrying LiDAR could be complex in the real world. Because of the uneven road or radical driving, LiDAR could be shaken suddenly, leading to jagged scan lines in LiDAR measurements. This kind of error brought about by motion can hardly be erased in the mapping process and would even affect semantic segmentation, object detection, filtering dynamic objects and so on. Some of the other methods attempt to reduce this impact by linear interpolation, ICP core, and so on. However, because of the existence of gimbal deadlock, spherical interpolation cannot be achieved using Euler angles and some did not make use of additional sensor data which reduce the processing accuracy.

In this study, we present a point cloud de-skewing algorithm to reduce the sensing error caused by ego-motion when mapping the surrounding area. Our algorithm integrates the high-frequency IMU data between two LiDAR scans to calculate the IMU motion discretely and treats IMU poses as optimization variables, restricted by LiDAR odometry. Then, interpolation is implemented to generate poses corresponding to every point. After transferring point clouds from the LiDAR frame to the IMU frame, the ceres solver is used to optimize the IMU poses until the de-skewing process converges. Compared with optimization in a single LiDAR scan, our algorithm can effectively eliminate the skewing by utilizing adjacent point clouds. Also, quaternion interpolation would not cause the risk of unpredictable problems.

The remainder of the paper is organized as follows. In Section 2, we summarize related works in the fusion of LiDAR and IMU measurements, point cloud registration and other de-skewing methods. In Section 3, we first illustrate LiDAR skewing brought by ego-motion and present an overview of our system. Then, we integrate IMU data and model interpolation, preparing for transformation. Before optimizing in ceres solver, LiDAR odometry is applied as a limitation in the following work. In Section 4, several experiments were performed to choose which ICP method and evaluate our de-skewing effect, including a discussion of them. Finally, we conclude our study in Section 5.

## 2. Related Works

There are primarily two kinds of approaches to deal with the fusion of LiDAR point cloud and IMU measurements. One of them is loosely coupled fusion that estimates the LiDAR and IMU respectively and then fuses the results. [9] uses a loosely coupled EKF (extended Kalman filter) to fuse IMU and 2D LiDAR but it cannot handle other complex environments. [10] presents a Multi-Sensor-Fusion Extended Kalman Filter to fuse IMU with other sensor data. It sacrifices the accuracy of the process results in exchange for computational efficiency as a result that it does not update the odometry part with IMU measurements. The other category, tightly coupled fusion, combines LiDAR and IMU measurements to estimate a common variable. [11] proposed a method to optimize the IMU measurements with the LiDAR odometry. [12] applies an error-state Kalman filter to tightly couple the IMU and LiDAR, updating corrections with the matched LiDAR heightmap and a prior DEM (digital elevation model). Although the tightly coupled method is more computationally complex than loosely coupled fusion, the former takes advantage of sensor data and gets better results.

3D point cloud registration is the front-end of the mapping pipeline and the registration consequences affect all the following mapping processes [13]. Point cloud registration is usually divided into two steps: coarse registration and fine registration. The first step is implemented to find out an approximate transformation when two point clouds are far from each other. LORAX divides the point cloud into many small pieces and generates descriptors using deep neural network [14]. 4-Points congruent sets [15] and its improved proposals [16,17,18] could register the point cloud quickly and robustly. The second one is to improve the accuracy after coarse registration, after having an approximate result. Fine registration is mainly processed with ICP (iterative closest point) and variants of ICP. The basic ICP pairs the corresponding points between two point clouds and evaluates the transformation to minimize RMS (root mean square) of all the point pairs. The algorithm needs an initial value and a suitable value could get better convergence. Point cloud segmentation and feature extraction can improve process efficiency and the quality of the registration results. However, this rudimentary method depends on a large amount of calculation and suffers from noise and outlier.

The LiDAR motion during its rotation period twists the point clouds since LiDAR rotates at a low frequency with 360° fields of view [19]. Once high acceleration motion occurs, the point clouds reflected by laser are disordered because of fast-moving. In [1], it preprocesses the LiDAR data to reduce distortion caused by the movement during the scan. It extracts roll, pitch and yaw from the rotation matrix between two scans. Then, the relative rotation of the point in the point cloud is approximated by linear interpolation and the rotation matrix, estimated from feature points, which is not accurate enough for de-skewing. [20] uses ICP core for de-skewing which does not make use of other sensor data. [21] only transfers the point clouds between two coordinate systems and has actually no de-skewing. [22] shows that skewing leads to trajectory drift when mapping the surrounding environment and exhibits visible rotation errors under the complex movement condition. It also attempts to de-skew with the assistance of high sampling IMU data but gives up eventually because of the challenging in calibration between LiDAR and IMU. [23] proposes a pre-processing module for de-skewing detailly. However, it interpolates Euler angles with linear interpolation which suffers from gimbal deadlock. [24] only conducted distortion correction at the odometry part which may cause a risk of cumulative error for later process.

## 3. Methods

### 3.1. The Impact of LiDAR Motion on Skewing

The frequency of LiDAR in the autonomous driving field is mostly under 20 Hz which is not very fast as opposed to cameras. Distance information measured by LiDAR is concerned with the flying time of the laser beam and firing channel. All these firing mechanisms, arranged vertically, rotates around the center of LiDAR and a scan period is the time of one rotation. The faster it rotates, the more information we get in a certain time but also shakes more heavily. In contrast, the slower, the more vulnerable to skewing. Considering the accuracy of measurement and the scan frequency of LiDAR, ranging from 5 Hz to 20 Hz, we set the frequency at 10 Hz.

**Figure 1:**
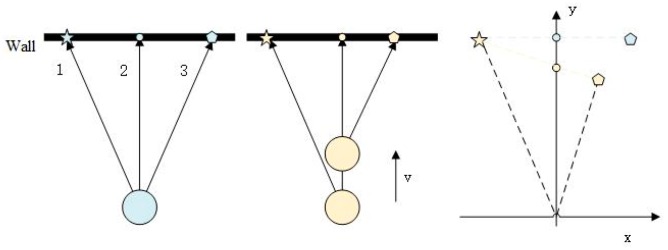
The effect of ego-motion on scan skewing.

**Figure 2:**
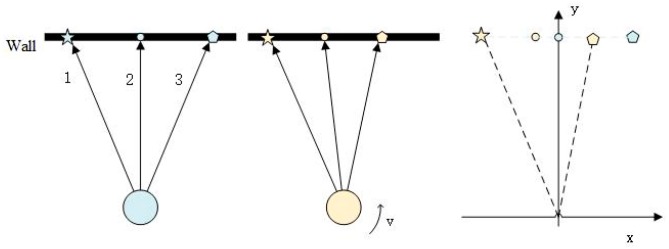
Effect of ego-rotation on scan skewing.

As we can see from the left part of Figure 1 and Figure 2, the LiDAR scan reflects the real wall correctly when LiDAR is stationary. Three scan points and the LiDAR itself constitute an isosceles triangle when LiDAR does not move or rotate. In order to find out the exact effects of LiDAR motion on scan skewing, we separate the motion into uniform linear motion and uniform circular motion for convenience and compare the point clouds with that under stationary condition. The middle part of two figures shows the LiDAR scan in two motion conditions and the right part of them shows the point clouds in two circumstances respectively. As we can see, linear motion mainly changes the vertical distance between the real obstacle and LiDAR itself while circular motion disorders the points in the horizontal distance, making the point cloud sparse or dense on each channel. Key skewing problems lie in the change of coordinate system, no matter which dimension, caused by ego-motion.

However, LiDAR motion in real experience is so complicated that skewing is also complex and cannot reflect the real information of the environment. The random motion of LiDAR could push or pull, stretch or compress the scan points. Also, the faster a LiDAR moves, the horrible the scanned point cloud becomes. Thus, a simple geometric method cannot handle these complex skewing since LiDAR vibration is usually unpredictable, leading to irregular point cloud skewing.

### 3.2. Overview of De-Skewing System

**Figure 3:**
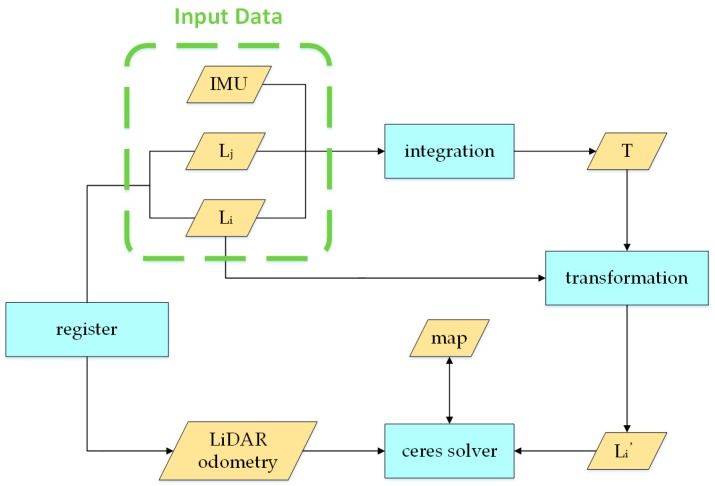
De-skewing system framework.

**Figure 4:**
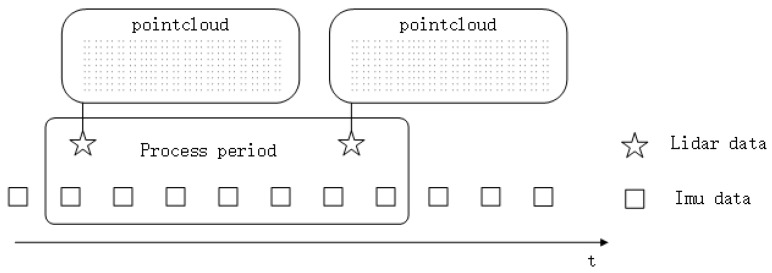
Sensor data in a processing period.

Figure 3 describes the workflow of our de-skewing algorithm. We fuse both point cloud and IMU data for the de-skewing process. Every process period contains two LiDAR point clouds and a series of IMU data during the scan period, as shown in Figure 4. The last LiDAR scan ${ℒ}_{i}$, in Figure 3, corresponds to the first point cloud in the processing period of Figure 4 and the current LiDAR scan ${ℒ}_{j}$ matches with the second one in Figure 4. The de-skewing process performs as follows: (1) Update IMU state iteratively when new data comes; (2) after receiving the LiDAR scan ${ℒ}_{i}$, integrate IMU data as *T* in Figure 3 to be used for transformation (Section 3.3); (3) at the time when the other point cloud ${ℒ}_{j}$ received, estimate the LiDAR odometry through registration (Section 3.4); (4) transform points from the LiDAR frame to the IMU frame and then, to the frame of the first point in the current point cloud as ${ℒ}_{i}^{\prime }$ in Figure 3 (Section 3.3); (5) setting the ceres solver to minimize the skewing problem with the assistance of a point cloud map (Section 3.5). The LiDAR odometry is utilized to reduce divergence results; (6) the current process is finished when reaching the number of iteration or the amount of change in the cost function is smaller than a threshold.

### 3.3. IMU Odometry and Coordinate Transformation

Measured data from IMU are in the form of the Euler angle and are transferred to quaternion when updating the IMU odometry. We denote the three-dimensional coordinates of IMU measurements as *p_i_* ∈ ${\mathbb{R}}^{3}$, where *i* represents the time sequence of the measurement stream. Similarly, quaternion *q_i_* ∈ ${\mathbb{R}}^{4}$, corresponding to the rotation matrix *R_i_*, and *v_i_* ∈ ${\mathbb{R}}^{3}$ are the orientation and velocity parts of IMU data. Conveniently, we take quaternion form of rotation for IMU odometry and the conversion from Euler rotation to *q_i_* and then to *R_i_* is shown as Equations (1) and (2)

(1) $$q_{i}=\left[\begin{array}{c} w\\ x\\ \begin{array}{c} y\\ z \end{array} \end{array}\right]=[\begin{array}{l} \cos\left(\frac{\varphi }{2}\right)\cos\left(\frac{\theta }{2}\right)\cos\left(\frac{\psi }{2}\right)+\sin\left(\frac{\varphi }{2}\right)\sin\left(\frac{\psi }{2}\right)\sin\left(\frac{\psi }{2}\right)\\ \sin\left(\frac{\varphi }{2}\right)\cos\left(\frac{\theta }{2}\right)\cos\left(\frac{\psi }{2}\right)-\cos\left(\frac{\varphi }{2}\right)\sin\left(\frac{\psi }{2}\right)\sin\left(\frac{\psi }{2}\right)\\ \cos\left(\frac{\varphi }{2}\right)\sin\left(\frac{\theta }{2}\right)\cos\left(\frac{\psi }{2}\right)+\sin\left(\frac{\varphi }{2}\right)\cos\left(\frac{\psi }{2}\right)\sin\left(\frac{\psi }{2}\right)\\ \cos\left(\frac{\varphi }{2}\right)\cos\left(\frac{\theta }{2}\right)\sin\left(\frac{\psi }{2}\right)-\sin\left(\frac{\varphi }{2}\right)\sin\left(\frac{\psi }{2}\right)\cos\left(\frac{\psi }{2}\right) \end{array}],$$

(2) $$R_{i}=\left[\begin{array}{ccc} 1-2q_{y}^{2}-2q_{z}^{2} & 2q_{x}q_{y}-2q_{z}q_{w} & 2q_{x}q_{z}+2q_{y}q_{w}\\ 2q_{x}q_{y}+2q_{z}q_{w} & 1-2q_{x}^{2}-2q_{z}^{2} & 2q_{y}q_{z}-2q_{x}q_{w}\\ 2q_{x}q_{z}-2q_{y}q_{w} & 2q_{y}q_{z}+2q_{x}q_{w} & 1-2q_{x}^{2}-2q_{y}^{2} \end{array}\right],$$

where *φ*, *θ*, *ψ* are the rotation angles around *x*, *y*, *z* axes and $q_{w},q_{x},q_{y},q_{z}$ are the four components of the quaternion *q_i_*, corresponding to *w*, *x*, *y*, *z* in Equation (1).

Every time when new IMU data arrives, we update the IMU state by discrete evolution [8] as Equations (3)–(5),

(3) $$p_{j}=p_{i}+\sum_{k=i}^{j-1}\left[v_{k}\Delta t+\frac{1}{2}g^{W}\Delta t^{2}+\frac{1}{2}R_{k}\left(a_{k}-b_{gk}\right)\Delta t^{2}\right],$$

(4) $$v_{j}=v_{i}+g^{W}\Delta t^{2}+\sum_{k=i}^{j-1}R_{k}\left(a_{k}-b_{ak}\right)\Delta t,$$

(5) $$q_{j}=q_{i}⊗\prod_{k=i}^{j-1}\delta q_{k}=q_{i}⊗\prod_{k=i}^{j-1}\left[\begin{array}{c} \frac{1}{2}\Delta t\left(\omega_{k}-b_{gk}\right)\\ 1 \end{array}\right],$$

in which *Δt* is the time between two consecutive IMU data and *g^W^* is the vector form of gravity in the world frame. *a_k_* and *ω_k_* are linear acceleration and angular acceleration of raw IMU measurements. *b_gk_* and *b_ak_* are gyroscope bias and acceleration bias respectively.

All the IMU data are integrated during the point cloud scan period, from *k* = *i* to *k* = *j*. Then, each IMU input corresponds with its relative pose in the LiDAR scan frame, which is optimized.

**Figure 5:**
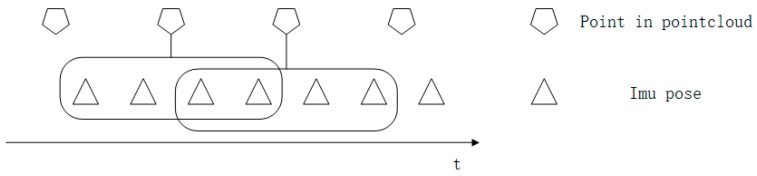
The pose of every point.

Although poses are integrated between every two consecutive IMU data, they do not correspond with points in the point cloud. In order to estimate the pose of point, interpolation is applied. The more interpolation variables are used, the more accurate the interpolation result is. However, this could result in a large amount of calculation and the interpolation results may become overfitting when the number of interpolation variables exceeds a certain threshold. As a result, the four closest poses, calculated by IMU data, are chosen for interpolation which balances the accuracy and efficiency, as shown in Figure 5. We represent location with a three-dimensional vector and rotation with quaternion. Each pose contains both location and rotation. Since quaternion has additional constraints, we interpolate location and rotation respectively.

#### 3.3.1. Location Modeling

We use Lagrangian interpolation to establish location function on time. It is used to fit the IMU motion trajectory by discrete measurements. Similar to the two-dimensional coordinate system, we replace the variable with a three-dimensional pose vector as Equations (6) and (7)

(6) $$L_{location}\left(t\right)=\sum_{j=0}^{n}p_{j}\ell_{j}\left(t\right),$$

(7) $$\ell_{j}\left(t\right)=\prod_{i=0,\;i\neq j}^{n}\frac{t-t_{i}}{t_{j}-t_{i}},$$

where *t* and *p_j_* are time and three-dimensional vector of IMU pose respectively and the number *n* is four as we choose the four closest IMU pose.

#### 3.3.2. Rotation Modeling

**Figure 6:**
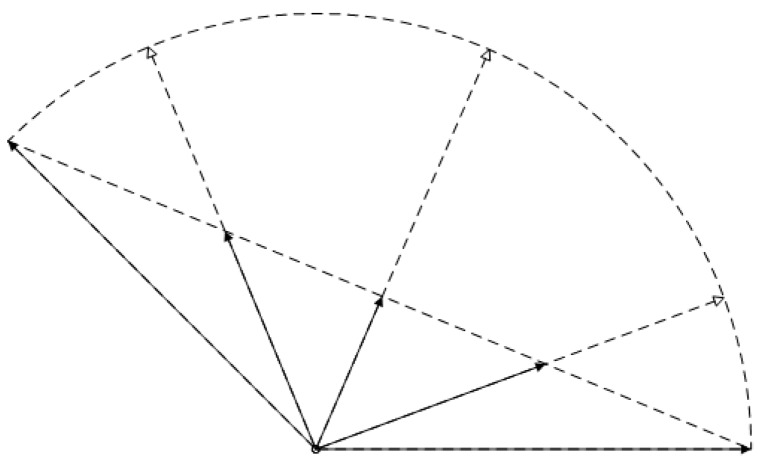
Linear orientation interpolation on arc length when interpolation over 1/4 intervals.

Because of the existence of gimbal deadlock, spherical interpolation cannot be achieved in Euler angles. Quaternion interpolation is not the same with any linear interpolation. Linear interpolation can hardly separate rotation evenly, as shown in Figure 6. The chord length is divided equally into four but the arc length is not separated evenly.

**Figure 7:**
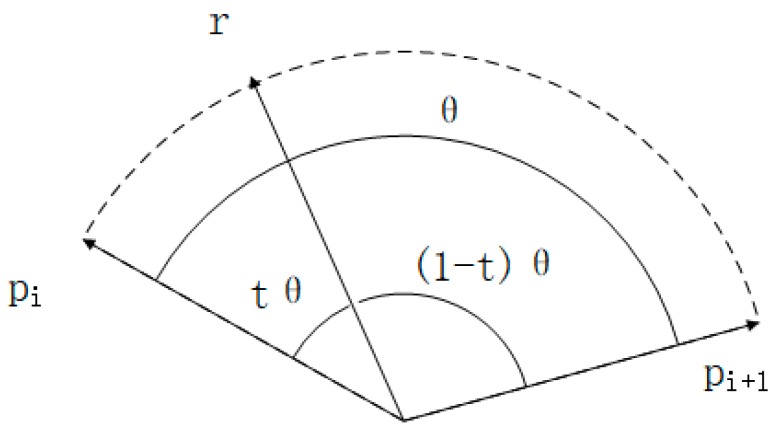
Spherical linear interpolation.

We use unit quaternion to represent rotation in this study, which signifies a four-dimensional hypersphere. Glenn Davis proposes slerp [25] (spherical linear interpolation) to interpolate between two quaternions, as Equation (8)

(8) $$r=\mathrm{slerp}\left(p_{i},p_{i+1},t\right)=\frac{sin\left(1-t\right)\theta }{sin\theta }p_{i}+\frac{\sin t\theta }{\sin\theta }p_{i+1},$$

where *p_i_* and *p_i+1_* are known IMU quaternion updating iteratively, *r* is the quaternion, the point rotation in the current scan, to be solved, and *t* is the interpolation variable, which ranges from 0 to 1, as shown in Figure 7.

However, when *r* comes close to *p_i_* or *p_i_*_+1_, slerp result is not accurate enough because we have not utilized other neighborhood rotation messages. To improve this, Dam and Erik B propose squad [26] (spherical and quadrangle) based on slerp,

(9) $$\begin{array}{c} \mathrm{squad}\left(p_{i},p_{i+1},s_{i},s_{i+1},t\right)=\mathrm{slerp}(\mathrm{slerp}\left(p_{i},p_{i+1},t\right),\\ \mathrm{slerp}\left(s_{i},s_{i+1},t\right),2t\left(1-t\right)), \end{array}$$

where auxiliary quaternion *s_i_* is taken as the temporary controller,

(10) $$s_{i}=exp\left(-\frac{log\left(p_{i+1}p_{i}^{-1}\right)+log\left(p_{i-1}p_{i}^{-1}\right)}{4}\right)p_{i},$$

and the interpolation variable *t*, as (8), the same as slerp, shown in Figure 7,

(11) $$t=\frac{t_{r}-t_{p_{i}}}{t_{p_{i+1}}-t_{p_{i}}},$$

*t_r_* is the timestamp of point in the current point cloud timeline, $t_{p_{i}},\;t_{p_{i+1}}$ are timestamps of IMU pose, the closest between *t_r_*, integrated from IMU data.

After estimating the quaternion of every point, we transfer them to the rotation matrix by Equations (1) and (2) since they are four-dimensional vector and cannot directly participate in the vector multiplication.

#### 3.3.3. Coordinate Transformation

**Figure 8:**
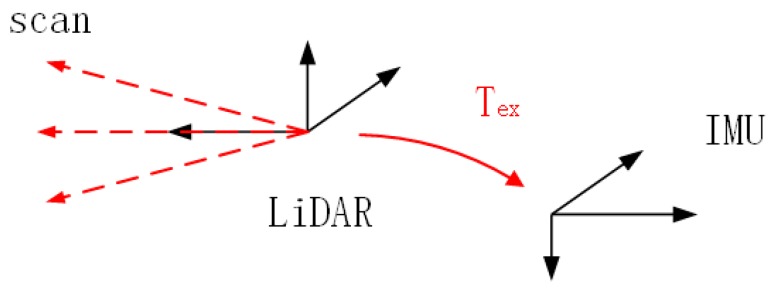
T_ex_ is the pose of inertial measurement unit (IMU) in the LiDAR frame, known from prior calibration.

As a result that LiDAR and IMU both have their own frame when collecting data, which is not convenient for further process, it is necessary to transform the point cloud from LiDAR frame to IMU frame by Equation (12), as shown in Figure 8.

(12) $$\begin{array}{c} x_{b}=R_{l}^{b}x_{l}+T_{l}^{b}\\ \left[\begin{array}{cc} x_{b} & 0\\ 0 & 1 \end{array}\right]=\left[\begin{array}{cc} R_{l}^{b} & T_{l}^{b}\\ 0 & 1 \end{array}\right]\left[\begin{array}{c} x_{l}\\ 1 \end{array}\right]\\ \left[\begin{array}{cc} x_{b} & 0\\ 0 & 1 \end{array}\right]=T_{ex}\left[\begin{array}{c} x_{l}\\ 1 \end{array}\right], \end{array}$$

The subscripts of *x_b_* and *x_l_* (*x* ∈ ${\mathbb{R}}^{3}$) represent the point data in IMU and LiDAR frame respectively. *R* is the rotation part of this transformation and the superscript and subscript denote the transform direction from the LiDAR frame to IMU.

Then, we transform all points of the current point cloud to the frame of the first point. Since location and rotation models are established already, we only have to determine the interpolate parameter *t* which concerns the time of IMU poses and LiDAR points.

We estimate the time of LiDAR points first. [27] describes the time management in each LiDAR scan in detail. Briefly, one data point period consists of three steps, laser firing, laser recharging, and data packing. Since the timestamp of every scan is the time of the first data point in the package, as shown in Figure 4, the time of each point in this scan *t_p_* could be calculated from the offset to the timestamp of the current scan as Equations (13) and (14)

(13) $$t_{p}=t_{s}+t_{off},$$

(14) $$t_{off}=50\left(\mu s\right)\times \left(n_{channel}-1\right)+3\left(\mu s\right)\times \left(n_{data}-1\right),$$

where *t_s_* and $t_{off}$ is the timestamp of the current scan and time offset relative to the first point. The time offset $t_{off}$ consists of measurement time and intervals between firing. Since all sixteen lasers being fired and recharged every 50 μs and the cycle time between firing being 3 μs, the measurement part and cycles part could be deduced from the index of each channel.

However, this *t_p_* is the relative time in this scan. For further calculation, whether transferring *t_p_* to ROS frame or transferring IMU time back to this relative frame works.

### 3.4. Point Cloud Registration and LiDAR Odometry

**Figure 9:**
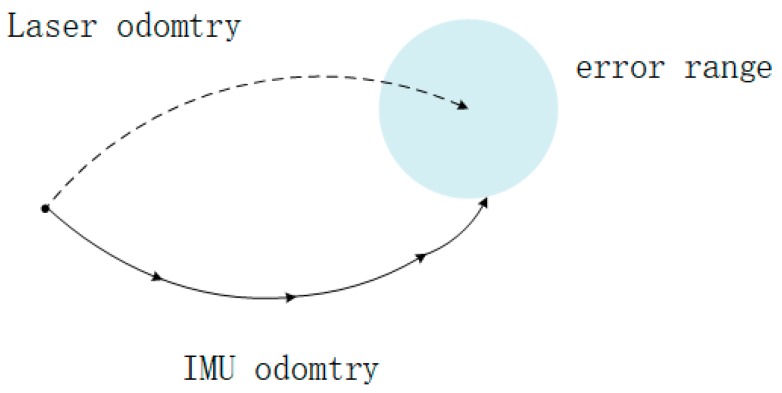
The error range in ceres solver.

Before optimizing in ceres solver, a limit shall be set in case of divergence, as shown in Figure 9. ICP algorithm is used to evaluate the rigid body transformation between two sets of point clouds. They are overlapped after one of them transforms into another through the transformation relationship solved by ICP. Pairs of corresponding points are chosen iteratively to calculate their distance which is used to refine the transformation by minimizing the sum of them. Because of the different definition of sampling, matching, weighting, rejecting, and error metric, this algorithm has various variants and are widely improved.

We use the PCL (point cloud library) in this study which is a large-scale, open project for 2D/3D image and point cloud processing. It contains numerous state-of-the-art algorithms and is convenient for adjustment parameters. Although the ICP algorithm has many variants, only some of them are available in PCL. Considering two point clouds are adjacent to two frames of LiDAR measurements, the initial matrix guess could be replaced with the pose integrated from IMU data.

### 3.5. Ceres Solver Optimization

For now, we have LiDAR odometry between two adjacent scans, point cloud to be de-skewed and discrete poses integrating from IMU measurements. However, poses interpolated from IMU may not be accurate enough for de-skewing since IMU data suffer from noise and bias. We try to optimize IMU poses through ceres solver to minimize the influence of measurement errors.

The optimization progress contains two tasks, one of which is to minimize the thickness of flat areas in a point cloud map and the plane function needs to be estimated first. Different from the idealized data set, the corresponding points of two consequent scans hardly exist, leading to the results of point cloud registration far from ground-truth. However, our approach only deals with points on a plane that are selected by the curvature and distance changes and have no requirement for strict correspondence. Every time when a new scan comes, it is transformed to a submap that only contains the point clouds near the LiDAR in a certain distance to reduce the computational complexity. Then all points in the submap are classified into different planes. In each plane, the plane function is fit by PCA and the IMU poses are adjusted to minimize the height difference in the plane area.

Plane fitting using PCA tries to find the normal vector $\vec{n}$ and the center of plane *q*,

(15) $$n^{⊺}\left(p_{i}-q\right)=0,\;p_{i}\in {ℒ}_{p},$$

where $p_{i}$ is the point in the plane area ${ℒ}_{p}$ and $n^{⊺}n=1$. While in real data, (15) can hardly be satisfied and thus it is replaced by minimizing the cost function below,

(16) $$\begin{array}{c} cost\left(n,q\right)=\underset{n^{⊺},q}{\min}\underset{i}{\sum }{\left|n^{⊺}\left(p_{i}-q\right)\right|}^{2}\\ =n^{⊺}A\left(q\right)A{\left(q\right)}^{⊺}n, \end{array}$$

where $A\left(q\right)≜\left[\cdots ,p_{i}-q,\cdots \right]$. The problem can be transferred as follows,

(17) $$\begin{array}{c} n^{*}=\arg\underset{n}{\min}n^{⊺}B\left(q^{*}\right)n\\ s.t.\;n^{⊺}n=1, \end{array}$$

After factorizing the $B\left(q^{*}\right)$ into the singular value decomposition, $B\left(q^{*}\right)=U\sum V^{⊺}$, the normal vector $\vec{n}$ is the last column of U and we get the point-normal equation for a plane,

(18) $$n_{x}\left(x-q_{x}^{*}\right)+n_{y}\left(y-q_{y}^{*}\right)+n_{z}\left(z-q_{z}^{*}\right)=0,$$

where $n_{x},n_{y},n_{z}$ are components of $\vec{n}$ in three axes, similarly as $q_{x}^{*},q_{y}^{*},q_{z}^{*}$. This can be simplified as,

(19) Ax+By+Cz+D=0,

and *A*, *B*, *C* are equal to $n_{x},n_{y},n_{z}$, $D=-\left(n_{x}q_{x}^{*}+n_{y}q_{y}^{*}+n_{z}q_{z}^{*}\right)$.

The residual for this part can be represented as,

(20) $$r_{ℒ}=\frac{1}{2}\sum_{p\in {ℒ}_{p}}\|Ap_{x}+Bp_{y}+Cp_{z}+D{\|}^{2},$$

**Figure 10:**
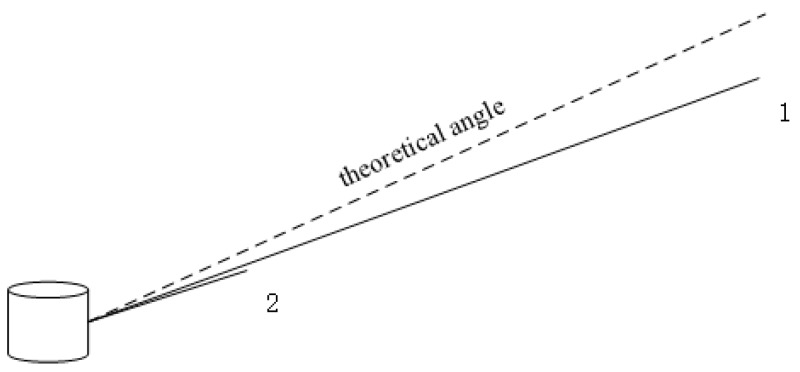
Impact of angle and laser path length on the degree of dispersion.

The other part of optimization is to minimize the degree of dispersion of points on the same scan channel. As a result that the distance between each measured point and LiDAR is different, points of each channel are not part of a common plane but a tapered surface. Thus, we think of angular deviation as an optimization variable. However, this single parameter cannot reflect the degree of dispersion objectively since the laser path length of different points is various, as shown in Figure 10. Although point 2 has a bigger angle gap to the theoretical angle than point 1, point 1 is far away from the theoretical line which is a severe error. The angular parameter is replaced by the distance to the tapered surface of the corresponding channel,

(21) $$r_{\Delta \theta }=\frac{1}{2}\sum_{p\in {ℒ}_{p}}\|l_{p}{\left(arctan\frac{p_{z}}{\sqrt{p_{x}^{2}+p_{y}^{2}}}-\varphi_{i}\right)\|}^{2},$$

*l_p_* is the laser path length and *φ_i_* represents the theoretical angle on the channel *i*. The channel information is unreachable in the usual PCL point cloud type and maybe is not published in some LiDAR driving program. Changing point type when programming or estimating the channel number through a vertical angle both can complete the missing information.

In order to avoid divergence result, we take trust region methods as the solving approach and reject the current optimization circle when the final IMU pose exceeds a certain range, defined by the odometry of LiDAR and IMU. The Levenberg-Marquardt algorithm is the most popular algorithm for solving non-linear least squares problems and was also the first trust-region algorithm to be developed. The LM method is an improvement based on the Gauss-Newton algorithm. By adjusting the parameters, the optimization can be freely switched between the gradient descent method and the Gauss-Newton method, and the fast convergence can be ensured while ensuring the drop. A common form of LM method is shown as,

(22) $$\left(J^{T}J+\lambda I\right)\delta =-J^{T}e,$$

where *J* is the gradient of the raw function relating to the independent variable, *I* is the identity matrix, *δ* is the amount of change in the independent variable, *e* is the error of raw function. The damping factor *λ* (non-negative) is adjusted at each iteration. A smaller value can be utilized to bring the algorithm closer to the Gauss-Newton algorithm or *λ* can be increased to give a step closer to the gradient-descent direction.

Because of the existence of measurement error and algorithm defect, the transformation evaluated from LiDAR odometry and IMU integration is different and we define the pose error between them in the first optimization circle as the error range, as the light blue area in Figure 9. In the following optimization, a result is discarded when the distance between transformation calculated from IMU poses and LiDAR odometry is larger than the error range.

## 4. Results and Discussion

In this section, we performed and analyzed several experiments. The first part, ICP registration comparison tried to find the best ICP method for indoor and outdoor experiments. Then, the latter two parts were conducted to evaluate our algorithm.

We received LiDAR point cloud and IMU measurements on the ROS (robot operating system) under the Linux system. The results of point clouds were presented in the PCL viewer, a third-party plugin. MATLAB was used to process data comparing with the ground-truth. Before the experiment, external calibration between LiDAR and IMU is evaluated. Similar to [11,28], we combined LiDAR odometry and IMU motions to solve the external parameters first which were optimized in ceres solver later.

### 4.1. ICP Registration Comparison

**Figure 11:**
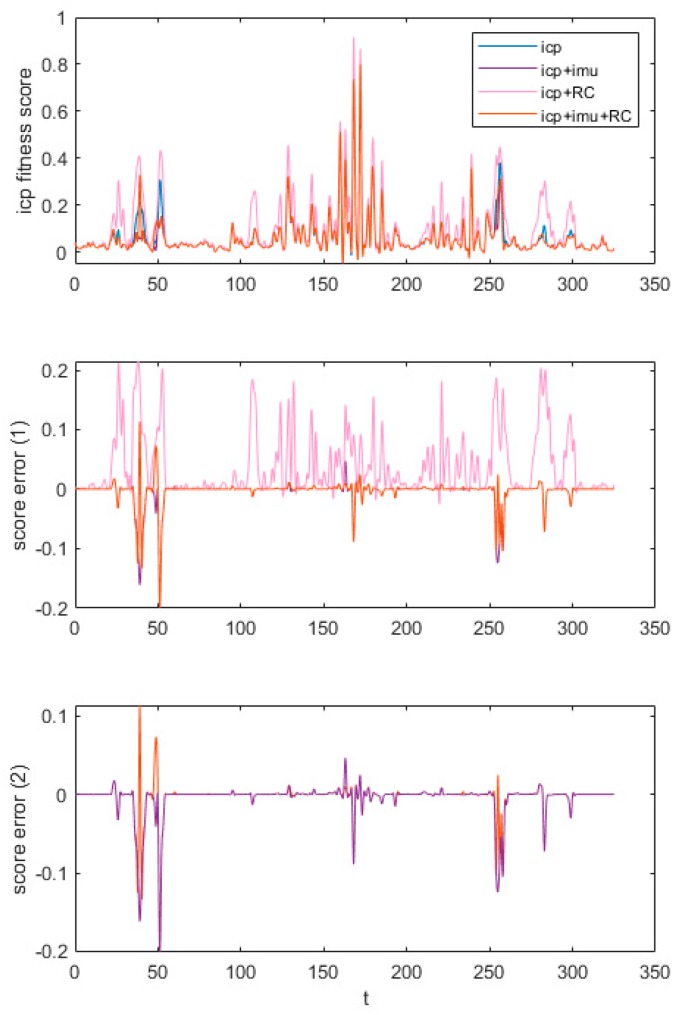
Comparison of different ICP registrations.

We compared the different configurations of the ICP register to find out the best parameter for the following experimental data set. Codes on the PCL document were taken as the basic ICP method, which was the first, blue one on the legend in Figure 11. Then, we added the IMU pose as the initial guess of the ICP algorithm. Reciprocal correspondences were added to the third method and the final one combined both the initial guess and reciprocal correspondences. We tested both indoor and outdoor data set and these methods showed little difference in the outdoor part. Thus, we focus on the indoor part.

We ignored the first and last 10% data to avoid the effect of severe motion. The first part of Figure 11 showed the ICP fitness score of four separate ICP configurations. The smaller the score, the better the result showed. The third method gets an extremely higher score sometimes. When it came to extreme points, this method scored the highest in all of them mostly. But other parts were similar to each other and can hardly tell the difference between them. Then, we set the first method, the basic ICP algorithm, as a reference and drew the score error curves between the reference and the rest three methods respectively, as shown in the middle part of Figure 11. As we can see, the pink one scored much higher than those two with IMU input as an initial guess. Thus, the ICP method with reciprocal correspondences was not the best choice. However, as a result that the curve plotted first could cover the latter one, it can hardly tell which method was better for the rest two of them. Then, we change the plot sequence as shown in the final part of Figure 11. Combining the last two parts of Figure 11, we find that both two methods performed similarly most of the time. While at some extreme points, the curve in purple had a lower score than that in orange. Above all, reciprocal correspondences did not improve the ICP performance a lot and the second method, ICP with IMU input, would become our registration program in the following experiments.

### 4.2. Indoor Experiments

**Figure 12:**
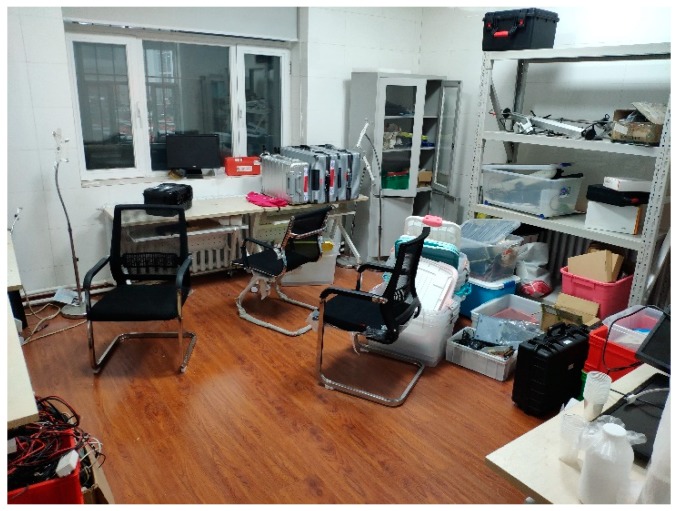
Scanned room in the indoor experiment.

In this section, we moved the pair of LiDAR and IMU around by hand in a room to construct the 3D information of the surrounding space, as shown in Figure 12. We held LiADR and IMU in the air first and kept them horizon, just like they were mounted on a vehicle. Then, we moved them in a circular route, the radius of which was about 20 cm. To model vibration in the real world, the LiDAR-IMU pair was rotated in various directions and artificial jitter was added occasionally to simulate sudden shaking when our vehicle went through a pit.

**Figure 13:**
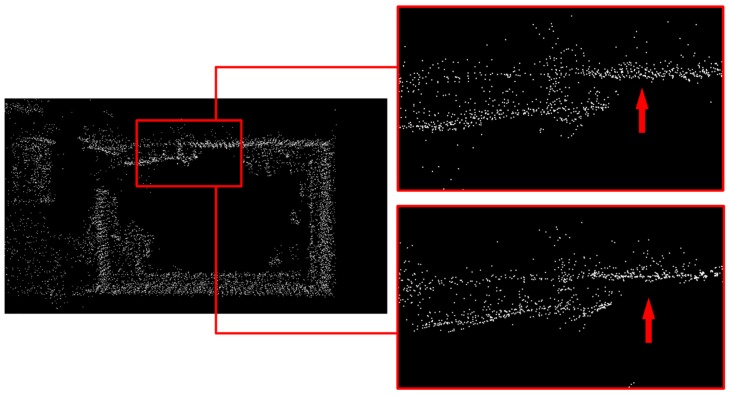
Cloud before and after de-skewing. The left part was the top view of our room. The above part on the right side of this figure represents the point cloud before de-skewing and the below part after de-skewing.

Figure 13 showed the mapping results of our room before and after de-skewing. Because of our room being in a mess, we chose a relatively clear wall for comparison, as the red rectangle in the left part of Figure 13. Then, we enlarged this area and demonstrated two pictures, representing raw point cloud and de-skewed point cloud respectively. As we can see from two red arrows, the point cloud of a wall after de-skewing rowed more orderly and arranged flatter than that before de-skewing. The “thickness” of the wall was also reduced. However, because of our mapping algorithm’s precision, there were still some messy points around.

**Figure 14:**
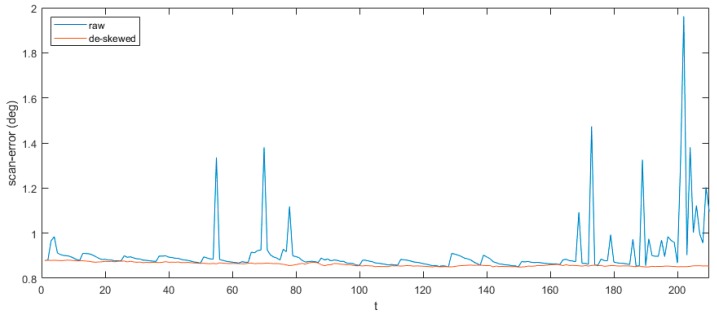
Errors changing by time.

Figure 14 and Table 1 showed exactly the error of the indoor experiment. We calculated the average angle errors in one scan changing with time and the RMSE of each channel. As we can see in Figure 14, the curve of raw data changed rapidly sometimes because of unstable motion. The sawtooth wave of raw data in blue resulted from our regularly rotating the LiDAR-IMU pair at random speed. After de-skewing, the scan error curve became smoother without massive pulse shapes. Overall, the orang curve stabilized around 0.8° which was lower than that of raw data. The ground truth of each channel’s vertical angle could be found in [27]. Sixteen scans are equally distributed in the vertical area from −15.0° to +15.0°. Table 1 showed the comparison of channel accuracy before and after de-skewing. RMSE in nearly all 16 channels showed a better performance after de-skewing. The data after de-skewing had an average 13.7% decrease. The gap between two sorts of numbers seemed smaller around 7 and 8 channels. However, it turned larger when it came to 0 and 15 channels.

### 4.3. Outdoor Experiments

The last two outdoor experiments were conducted on the school campus. The former one aimed to find out whether our approach would reduce the effect of the deceleration zone and the latter experiment was taken place on the uneven road.

**Figure 15:**
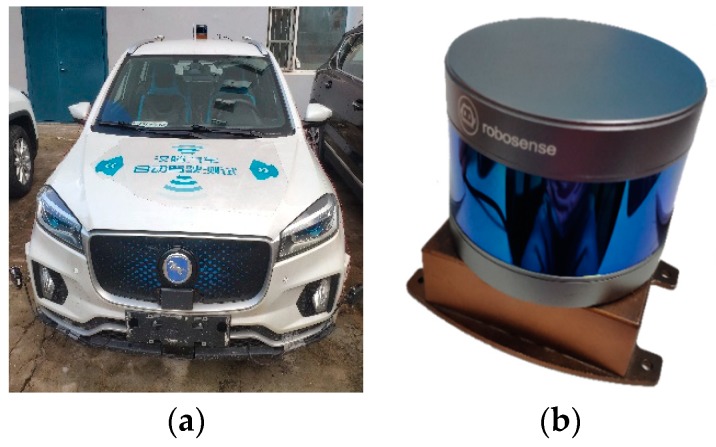
(**a**) Experimental platform; (**b**) LiDAR-IMU pair.

Our experimental platform was an autonomous vehicle, as shown in Figure 15a. 16-line 3D LiDAR and IMU were mounted on the top of the vehicle. The rotation frequency of LiDAR had three different modes: 5 Hz, 10 Hz, and 20 Hz. A lower frequency achieved a higher angular resolution while a higher frequency could get more real-time information. Considering the accuracy and timeliness, our LiDAR rotation frequency was set to 10 Hz. Different from LiDAR, IMU updates measurements in a much higher frequency, up to 1000 Hz. However, a higher frequency might lead to uncertain errors of communication between IMU and a computer. After comparing communicating stability under various frequencies, we finally set our IMU frequency at 100 Hz. In order to reduce the relative movement, the LiDAR and IMU were placed closely and fixed together, as shown in Figure 15b. Also, a high precision RTK-GPS, mounted at the trunk, was used to provide the ground-truth of poses in the outdoor experiment. During the outdoor experiment, the estimated trajectories are aligned with the ground truth in [29].

**Figure 16:**
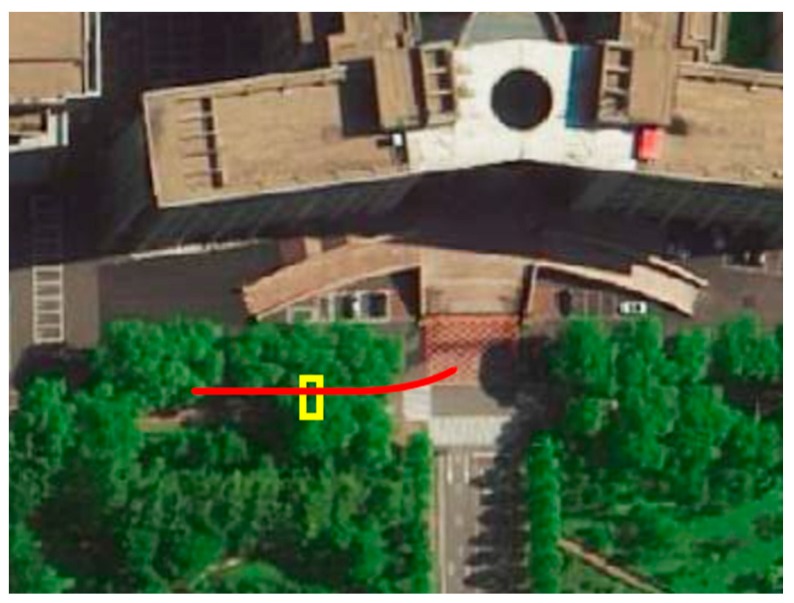
The real scene of outdoor experiment location. The red line represents the driving route of our vehicle and the yellow rectangle is the place of the deceleration zone.

The first experiment is performed in front of a building to show the performance of our method when the vehicle going through a deceleration zone, as shown in Figure 16. We drove the vehicle along the red route to rebuild the environment information. We started at the center of the crossroad and drove at a speed of 15 km/h under those trees. After passing through the deceleration zone, the yellow rectangle in Figure 16, we decelerated till our vehicle stopped.

**Figure 17:**
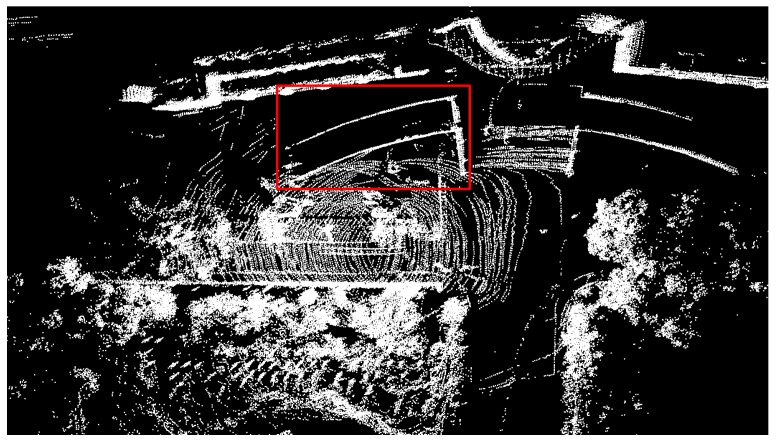
Rebuilt environment in which white points are laser reflection points. The red rectangle area is going to be compared later in Figure 18.

**Figure 18:**
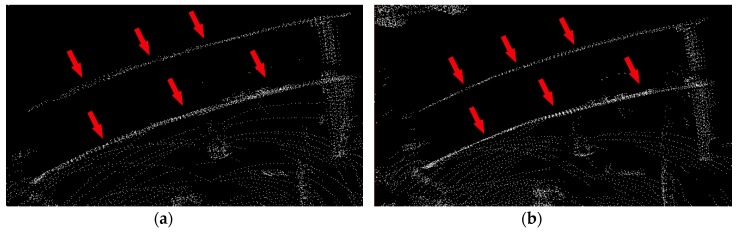
Clouds represent the red comparison area in detail in Figure 17. Two walls were pointed out by red arrows. (**a**) Represents the raw point cloud; (**b**) is processed with our de-skewing approach.

The environment rebuilt by our algorithm is shown in Figure 17. For visual comparison, we choose an area with two parallel walls, as the red rectangle area shown in Figure 17. We focused on the point clouds in this area to evaluate the performance of our method because flat walls were the best, obvious area for comparison and these two walls were the nearest and clearest. Also, these two walls had different height. The above one in the red rectangle area was taller than the below one so that we could see both of them at the same time. To show the red rectangle area more clearly, we amplified this area and compared two point clouds before and after de-skewing, as shown in Figure 18. The left picture was the raw point cloud and the right one was after de-skewing. After comparison, the point cloud of two walls on the right side of Figure 18 was thinner, denser than that on the left side. Also, two walls looked brighter after de-skewing which meant that points were gathered more closely.

**Figure 19:**
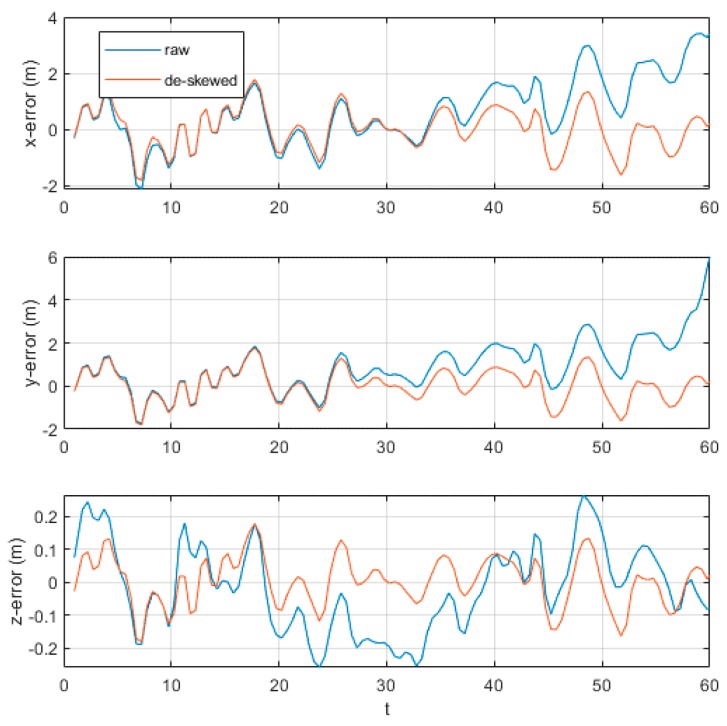
Errors in the outdoor test.

We input a high accuracy GPS signal as the ground truth to evaluate the trajectory errors. Figure 19 showed the trajectory errors in three dimensions. Whether with or without de-skewing, pose errors of two kinds were highly related. Both two curves of x-error and y-error in the first half shared little difference. However, when it came to the second half, the gap between them became wider. The trajectory in x and y dimensions of de-skewed point clouds was closer to ground truth and showed less drift. Although z-error in both conditions has similar extremum, the de-skewed curve was more stable than the raw curve.

The results of the accuracy evaluation are shown in Table 2 and Table 3. Pose evaluation showed that all six dimensions of the pose after de-skewing got a lower RMSE, compared with that without de-skewing, except roll dimension. Detailly, RMSE in x dimension decreased by about 41% and in y, z dimension, their RMSE both remained nearly half of them after de-skewing. Although the roll dimension had a little increase, three angular dimensions showed little difference, compared with the three locational dimensions. In channel evaluation, we added the maximum degree error as another evaluation index which represented the difference between the theoretical angle and the measured angle in each channel. The results in Table 3 showed that all 16 channels achieved better performance whether in RMSE or MAX after de-skewing. While in RMSE, channel 0 and 1 got worse consequences than channel 14 and 15. It was because, with the channel index increasing from 0 to 15, the corresponding vertical angle decreased from +15.0° to −15.0°. As a result, most lasers in channel 0 and 1 were fired upwards which might be easily out of the effective range of our LiDAR scan. Thus, these over ranged points showed worse performance than those fired downwards.

**Figure 20:**
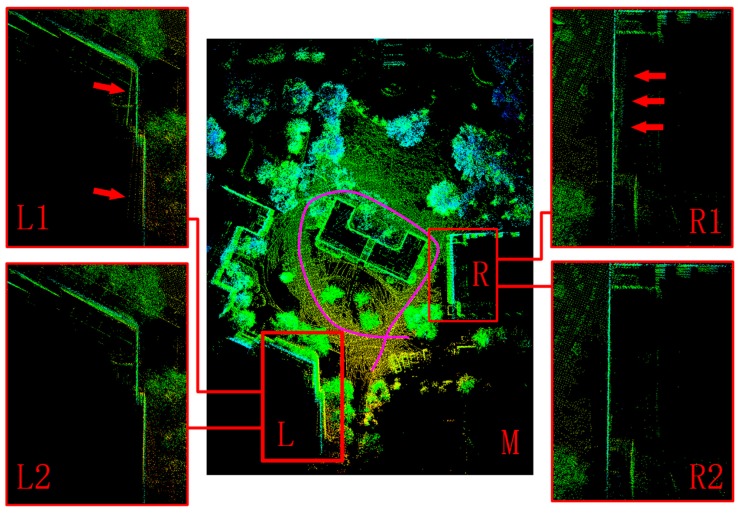
Outdoor experiment on a larger scale. The pink curve was the driving route. Different colors represent different reflection intensities of points. M: the scanned area, with two comparison areas L and R in red rectangles; L1: enlarged view of L without de-skewing; L2: enlarged view of L with de-skewing; R1: enlarged view of R without de-skewing; R2: enlarged view of R with de-skewing.

The second part of the outdoor experiment was performed in a larger scan area. because of the long-term use, this section of the road was quite uneven and had many cracks or even some gravels and pits on the surface. We drove along the pink route in a counterclockwise direction at a speed of 25 km/h. Similarly, we calculated the channel accuracy in this part. A significant improvement was seen after de-skewing in both RMSE and MAX, as shown in Table 4. RMSE showed an average of ten times decrease after de-skewing. Because of the tough road conditions, data of RMSE and MAX before de-skewing were much larger than that of the former part in the outdoor experiment, while our method still achieved a similar error level. The mapping result is presented in Figure 20. We chose two large wall areas for evaluation: L and R. We took a look at L first. Before de-skewing, some shadow points of the wall were highlighted by two red arrows in L1. When one wheel of our vehicle went into a pit, the sudden vibration transferred to LiDAR, leading to these lines. While after de-skewing, L2 showed few lines in the same place. Then, the R area was another building. As shown in R1, three red arrows point out disorganized points behind the wall caused by many cracks in front of this building. After de-skewing, this wall became more clear.

### 4.4. Discussion

The main contribution of this study is to develop a de-skewing system by fusing de-skewing into the mapping process and estimating the pose of points with quaternion interpolation. Before the formal experiment, ICP configuration was first confirmed to adapt to different experimental environments. We conclude that with IMU input as an initial guess, ICP achieved the best performance. Both indoor and outdoor experiments showed that the mapping errors affected by skewing were greatly reduced. In the indoor experiment, point clouds became more ordered and the average angle errors were more stable and less after de-skewing. Also, in the outdoor experiment, the first part demonstrated clearer point clouds of walls after de-skewing. Two trajectory errors, x and y, showed a high relationship when changing with time. However, after going through the deceleration zone, at about 30 s, the curve with de-skewing showed less error. In addition, the trajectory error in the z dimension changed a lot whose maximum was over 0.2 m, while our vehicle was driving on the flat road. That would result from our unsatisfactory mapping system, showing weakness in estimating the z dimension. In the second part, flat walls became clearer and showed a little shadow of walls caused by skewing, compared with those without de-skewing. Although maximum channel errors were much larger than those in the former part, they finally reduced to a similar degree after de-skewing.

## 5. Conclusion

This study presents a LiDAR de-skewing method which takes advantage of IMU measurements to de-skew LiDAR point clouds. We integrate the high-frequency IMU data between two LiDAR scans to calculate the IMU motion discretely and optimize both the point cloud and IMU data in ceres solver. Our method mainly made two contributions: (1) After updating the IMU state by discrete evolution, we apply Lagrangian interpolation and spherical and quadrangle to modeling the pose function in a certain period. Thus, we avoid the problem of gimbal deadlock and achieve better efficiency. (2) In the optimization process, the scan to be optimized is de-skewed in a submap rather than in a single point cloud. The extra information in the submap helps improve the accuracy of de-skewing results. All potentially available point clouds have maximized their effectiveness.

Our method was evaluated in various experiments. First of all, the best parameter of the ICP method was selected by conducting an extra experiment and we found that reciprocal correspondences did not improve the ICP performance a lot. However, the second method, ICP with IMU input, performed the best among them. Afterward, both the results of indoor and outdoor experiments show that our algorithm improves the quality of point clouds and smooths the trajectory errors at the time of mapping. Skewing point clouds resulting from a sudden rapid movement are greatly corrected after optimization, especially those highlighted walls. Compared with original data, RMSE and MAX in each channel decreased by 13.7% and 71.0% respectively. Also, RMES in trajectory evaluation dropped over 30%.

Further work on our study can focus on limiting the processing time to improve efficiency. As a result that one LiDAR scan contains lots of points, the optimization period takes considerable computational time to adjust the transformation of each point. This reflects the low efficiency of our algorithm in a certain respect. Furthermore, in the first part of our outdoor experiment, trajectory error in the z dimension performs badly because of our immature mapping method. Thus, the accuracy of our mapping process can be improved greatly.

## Figures and Tables

**Table 1:** Evaluation of channel accuracy in the indoor experiment.

Channel	RMSE (No De-Skewing)	RMSE (After De-Skewing)	Channel	RMSE (No De-Skewing)	RMSE (After De-Skewing)
0	1.7895	1.5708	8	0.1405	0.0996
1	1.5165	1.3302	9	0.3232	0.2780
2	1.2540	1.1161	10	0.5124	0.4482
3	1.0288	0.9084	11	0.7154	0.6290
4	0.7912	0.6937	12	0.9312	0.8226
5	0.5679	0.4946	13	1.3025	1.1915
6	0.3453	0.2949	14	1.5685	1.4473
7	0.1411	0.1008	15	2.5504	2.3487

**Table 2:** Pose accuracy in the outdoor experiment.

Pose	RMSE (No De-Skewing)	RMSE (After De-Skewing)
X	1.5750	0.9257
Y	1.9159	0.9645
Z	0.1436	0.0741
Roll	0.0096	0.0103
Pitch	0.1010	0.0895
Yaw	0.0935	0.0792

**Table 3:** Channel accuracy in the outdoor experiment.

Channel	RMSE		MAX (deg)	
	No De-Skewing	After De-Skewing	No De-Skewing	After De-Skewing
0	0.5201	0.3499	2.8023	0.358
1	0.1748	0.1591	0.3624	0.1643
2	0.1089	0.0959	0.2811	0.0984
3	0.0745	0.0658	0.1996	0.0688
4	0.0448	0.0374	0.1374	0.0395
5	0.0446	0.0398	0.1092	0.0441
6	0.0484	0.0458	0.089	0.0479
7	0.0086	0.0073	0.0274	0.0084
8	0.0099	0.0104	0.0115	0.011
9	0.0174	0.0130	0.0661	0.0145
10	0.0372	0.0308	0.122	0.037
11	0.0587	0.0500	0.1747	0.0573
12	0.0827	0.0720	0.2288	0.0840
13	0.1272	0.1146	0.2961	0.1315
14	0.1322	0.1166	0.3241	0.1391
15	0.1415	0.1233	0.3637	0.1451

**Table 4:** Channel accuracy in the outdoor experiment.

Channel	RMSE		MAX (deg)	
	No De-Skewing	After De-Skewing	No De-Skewing	After De-Skewing
0	1.3610	0.1255	6	0.1299
1	1.1805	0.1088	5.2	0.1112
2	0.9990	0.0921	4.4	0.0948
3	0.8176	0.0753	3.6	0.0775
4	0.6377	0.0586	2.8	0.0643
5	0.4590	0.0419	2	0.0513
6	0.2838	0.0255	1.2	0.0299
7	0.1020	0.0093	0.4324	0.0134
8	0.1413	0.0112	0.8889	0.0169
9	0.4551	0.0348	2.1818	0.048
10	0.5982	0.0516	3.2	0.0667
11	0.8019	0.0718	2.9474	0.0913
12	1.0547	0.0940	4.1143	0.1137
13	1.2791	0.1179	5.5	0.147
14	1.5278	0.1449	6.1176	0.2023
15	1.8948	0.1764	7.5	0.2572
